# Genome-scale analysis of positional clustering of mouse testis-specific genes

**DOI:** 10.1186/1471-2164-6-7

**Published:** 2005-01-19

**Authors:** Quan Li, Bernett TK Lee, Louxin Zhang

**Affiliations:** 1 Institute for Infocomm Research, Heng Mui Keng Terrace 21, Singapore; 2 Department of Biochemistry, National University of Singapore, MD 7, Medical Drive, Singapore; 3 Department of Mathematics, National University of Singapore, 2 Science Drive 2, Singapore

## Abstract

**Background:**

Genes are not randomly distributed on a chromosome as they were thought even after removal of tandem repeats. The positional clustering of co-expressed genes is known in prokaryotes and recently reported in several eukaryotic organisms such as *Caenorhabditis elegans*, *Drosophila melanogaster*, and *Homo sapiens*. In order to further investigate the mode of tissue-specific gene clustering in higher eukaryotes, we have performed a genome-scale analysis of positional clustering of the mouse testis-specific genes.

**Results:**

Our computational analysis shows that a large proportion of testis-specific genes are clustered in groups of 2 to 5 genes in the mouse genome. The number of clusters is much higher than expected by chance even after removal of tandem repeats.

**Conclusion:**

Our result suggests that testis-specific genes tend to cluster on the mouse chromosomes. This provides another piece of evidence for the hypothesis that clusters of tissue-specific genes do exist.

## Background

Positional clustering of co-expressed genes is mainly due to operons in prokaryotes. Genes located within the same operon are transcribed together and thus co-regulated. In general, operons are missing from eukaryotes. Eukaryotic genes appear to be transcribed individually and are thought to be scattered on chromosomes without apparent organization by function or positional expression except for tandem duplication. However, positional clustering has recently been reported in several eukaryotes such as *Saccharomyces cerevisiae *[1,23], *Drosophila melanogaster *[4,5], *Caenorhabditis elegans *[2,3,7], and *Homo sapiens *[6,8].

Cho *et al*. first showed clustering of co-expressed yeast genes on a genome-scale. They found that about 25% of genes with cell-cycle-dependent expression pattern were adjacent to those induced in the same phase of the cell cycle [23]. This fact was also reported by Cohen *et al*. [1]. Cohen *et al*. computationally analyzed the whole-genome gene expression using the same dataset. They consider two ORFs to be adjacent if they occur on the same chromosome and if there are no other ORFs or functional elements such as Ty element, long terminal repeats, tRNAs, snRNAs between them. They observed that adjacent or nearby non-adjacent pairs of genes showed correlated expression independent of their orientation in the yeast genome using chromosome correlation maps that display correlations between the expression patterns of genes on the same chromosome. In addition, they also showed that genes with similar functions tend to occur in adjacent positions along the chromosomes.

Spellman *et al*. [4] found that numerous clusters that span 10 to 30 physically adjacent genes share strikingly similar expression profile in *Drosophila melanogaster *and these clustered genes account for over 20% of the total assayed genes. By mapping Expressed Sequence Tags (EST) back to the *Drosophila *genome, Boutanaev *et al*. [5] observed almost one thirds of 1661 testis-specific genes are clustered on chromosomes.

Similarly, positional clustering of co-expressed genes was also reported in *Caenorhabditis elegans*. Although operon and tandem duplication are major mechanisms for the observed co-expression gene clusters in the worm, co-expression gene clustering is still evident after exclusion of these two causes [3,7].

Clusters of highly co-expressed genes were also revealed in the human and other mammalian genomes [6,8,5,12]. Most of these studies focused on a set of tissue-specific genes that are highly expressed. For example, by analyzing the expression profiles of genes in carious tissues, Caron *et al*. [8] showed that highly expressed carious genes tend to cluster in large domains, called RIDGEs (*Region of IncreaseD Gene Expression*). However, these RIDGES seem to consist mainly of housekeeping genes [6]. Gabrielsson *et al*. [9] performed a microarray analysis of genes expressed in the human adipose tissue. By mapping these genes back to the human genome, they observed clusters of adipose tissue specific genes. Dempsey *et al*. [10] investigated genes from chromosomes 21 and 22 expressed in the human cardio-vascular system. Using EST (*Expressed Sequence Tag*), they showed positional clustering of these genes. These studies suggest that clusters of tissue-specific genes do exist, and might be more frequent than initially thought.

Here we performed a genome-wide analysis of testis-specific genes in the mouse genome using a method similar to one proposed in [5]. We used a publicly available EST (Expressed Sequence Tag) database to identify differentially expressed genes. After mapping testis-expressed ESTs back to the genome, we obtained the testis-expressed gene expression profile by assembling overlapped ESTs. In the same way, we got embryo-expressed gene expression profile. Testis-specific genes were generated by subtracting embryo-expressed gene expression profile from testes-expressed one. From the testis-specific gene expression profile, clustered genes were observed.

To further investigate testis-specific gene clusters in the mouse genome, we used the full-length cDNA sequences publicly available in the Fantom 2.1.1 database to identify differently expressed genes. After mapping 60,770 cDNA sequences back to the genome, we obtained the gene expression profile by assembling overlapping cDNA sequences into a single gene model. Testis-specific genes were selected by using only sequences from libraries where the tissue type included the keyword "testis" and not the keyword "embryo". Again, our statistical analysis shows the existence of testis-specific gene clusters along the mouse chromosomes even after removal of tandem duplicated genes. This suggests that considerable clusters of tissue-specific genes do exist in higher eukaryotes. Our results provide further support for the hypothesis that chromatin domain model may be involved in the regulation of gene expression in higher eukaryotes

## Results

The testis-expressed profile is a global view of gene expression and provides a foundation for understanding how the tissue functions in molecular level. We assume that most of the testis-specific genes are expressed after embryo phases in mouse. Therefore, we selected only testis genes models that exclude any expression in embryo for the construction of the testis-specific gene profile, which reveals positional clustering along chromosomes. Here, we analyzed the cluster distribution on all the chromosomes except for chromosome Y. (In Y chromosome, we only identified 12 testis-specific genes and 7 other genes.) Based on our study, we obtain the following observations.

### a. Density of testis-specific genes

The testis-specific gene expression profile along the mouse genome shows that testis-specific genes are distributed on all the chromosomes with different gene density. The ratio (R) of the number of the observed testis-specific genes (O) to the expected number (E) is used to measure the gene density, where E is calculated according to the chromosome's size on assumption that the testis-specific genes are uniformly distributed throughout the genome. The lowest gene density appears on chromosomes 1 (R = 0.834), 15 (R = 0.893), 16 (0.855), and 18 (R = 0.767) except for sex chromosomes. But, chromosomes 9 (R = 1.145), 11 (R = 1.374), 14 (R = 1.207) and 19 (R = 1.48) have rather high gene density. Within a chromosome, the distribution of testes-expressed genes also shows diverse density.

### b. Genomic location is highly correlated with testis-specific gene expression

We observed numerous groups of physically neighboring testis-specific genes that shared strikingly similar expression levels. In eukaryotes, it is clear that the activity of a promoter can be modified by transcription factors binding to DNA sequence, which are located from hundreds to thousands of base pairs from the promoter. Recently, increasing evidences show that genes may also be regulated in a group. For example, when transgenes are removed from their local environment and reinserted elsewhere in the same genome, they tend to show variant levels of expression [13]. More commonly, most eukaryotic chromosomes exhibit transcriptionally active (euchromatin) or inactive (heterochromatin) regions. In animals, heterochromatin is typically found near the centromere and other regions of low sequence complexity. Since RIDGEs do exist and neighboring genes show similar expression level, we conclude that genomic location has impact on gene expression.

### c. A large proportion of testis-specific genes are in adjacent pairs

We consider two genes are adjacent if there are no other genes between them on the same chromosome. We observed that 1170 testis-specific genes appear in adjacent pairs, which account for 31.8% of the testis-specific genes after removal of tandemly duplicated genes. There are 350 adjacent pairs, 86 adjacent triplets, 23 adjacent quadruplets, and 4 quintuplets (see Table 1 for details), significantly more than would be expected by chance. In fact, the number of observed adjacent gene clusters (including singletons) is smaller than the expected number minus the standard deviation for every chromosome other than chromosomes 15 and 19.

**Table 1:** Chromosomal distribution of adjacent testis-specific genes after removal of tandemly duplicated genes.

Size	Cluster distributions of adjacent testis-specific genes on chromosomes																				
	
	**1**	**2**	**3**	**4**	**5**	**6**	**7**	**8**	**9**	**10**	**11**	**12**	**13**	**14**	**15**	**16**	**17**	**18**	**19**	**X**	Total
**2**	26	22	21	26	29	22	17	20	14	24	19	18	18	20	6	15	13	9	9	12	350
**3**	2	5	3	6	6	4	4	4	12	4	8	1	2	5	5	4	3	3	1	4	86
**4**	2	1			1	2	2	3	1	2	1	2	3	2						1	23
**5**						1						1		1						1	4

The genes in the quintuplets are probably worthy to be investigated biologically. There is a quintuplet on each of chromosomes 6, 12, 14 and X. The quintuplet on chromosome 6 is located in a 400 kb segment starting at 144691608 and ending at 145141155; the one on chromosome 12 is located in a 600 K segment starting at 101700908 and ending at 102297618; the one on chromosome 14 is located in a 700 K segment starting at 62802300 and ending at 63500814. However, the one on chromosome X seems less significant, which is located in a 3719 K segment starting at 113253694.

### d. Testis-specific genes show obvious clustering

We evaluated the positional clustering of testis-specific genes using a model called the neighborhood model. We define clusters using the distance between two neighboring testis-specific genes along the chromosome. Two testis-specific genes are in a cluster if there is a series of testis-specific genes between them such that the distance between two neighboring genes in the series is less than a specified distance (D). We obtained 3679 testis-specific genes on all the chromosomes except for Y after removal of tandemly duplicated genes. We conducted statistical analysis with 4 different values of D: 25 K, 50 K, 75 K, and 100 K (see Table 2).

**Table 2:** Distribution of testis-specific gene clusters after removal of tandemly duplicated genes. Here only the numbers of clusters having at least 2 genes on all the chromosomes except for chromosome Y are given.

Size	Cluster distributions on chromosomes (distance = 25 K)																				
	
	**1**	**2**	**3**	**4**	**5**	**6**	**7**	**8**	**9**	**10**	**11**	**12**	**13**	**14**	**15**	**16**	**17**	**18**	**19**	**X**	Total
**2**	18	22	17	22	23	13	17	17	18	22	16	12	15	15	6	8	7	7	7	7	288
**3**			1				1		2	1	2	3	1	2	1	3			1		21
**4**	1	1																			2
**5**																					
**6**																					

Size	Cluster distributions on chromosomes (distance = 50 K)																				
	
	**1**	**2**	**3**	**4**	**5**	**6**	**7**	**8**	**9**	**10**	**11**	**12**	**13**	**14**	**15**	**16**	**17**	**18**	**19**	**X**	Total

**2**	26	30	22	32	34	20	21	22	19	31	22	16	16	20	15	14	12	8	6	6	392
**3**			2	5	1	2	6	1	8	3	6	3	3	3	4	3	2	1	2	1	56
**4**	1	5	2				1	2			2	1	1	1					1	2	19
**5**													1								1
**6**																					

Size	Cluster distributions on chromosomes (distance = 75 K)																				
	
	**1**	**2**	**3**	**4**	**5**	**6**	**7**	**8**	**9**	**10**	**11**	**12**	**13**	**14**	**15**	**16**	**17**	**18**	**19**	**X**	Total

**2**	27	28	23	37	47	29	24	29	17	34	29	17	18	24	16	16	19	11	9	10	454
**3**	1	4	4	5	4	5	9	2	9	2	7	3	3	6	5	4	1	1	3	1	79
**4**	2	6	3				1	3	1	2	4	1	1	1			1		1	1	28
**5**		1							2				1								4
**6**											1										1

Size	Cluster distributions on chromosomes (distance = 100 K)																				
	
	**1**	**2**	**3**	**4**	**5**	**6**	**7**	**8**	**9**	**10**	**11**	**12**	**13**	**14**	**15**	**16**	**17**	**18**	**19**	**X**	Total

**2**	32	33	24	31	42	33	28	27	19	39	23	21	21	25	16	15	24	9	9	12	463
**3**	2	6	4	7	8	6	12	8	12	3	11	2	4	7	4	6	2	3	6	1	114
**4**	3	6	5	3	1	1		3	1	3	7	2	2	2	2	1	1		2	1	46
**5**		1		1			1		2				1		1						7
**6**											1			1							2

When D = 25 K, we obtained 288 two-gene, 21 three-gene, and 2 four-gene clusters. In total, 18% of the testis-specific genes are contained in these clusters. By incorporating the variance of gene density in different regions on a chromosome, we conclude that 45 two-gene, 7 three-gene and 2 four-gene clusters are significant with p-value less than 4.9·10^-2^, 3.1·10^-2 ^and 1.2·10^-2 ^respectively. The most significant cluster is the four-gene cluster on chromosome 2 (p-value = 1.3·10^-3^).

When D = 50 K, we obtained 392 two-gene, 56 three-gene, 19 four-gene and 1 five-gene clusters. In total, 28% of the testis-specific genes are in these clusters. By incorporating the variance of gene density in different regions on a same chromosome, we conclude that 14 two-gene, 6 three-gene and 2 four-gene clusters are significant with p-value less than 4.9·10^-2^, 3.1·10^-2 ^and 1.2·10^-2 ^respectively. The most significant one is a two-gene cluster on chromosome 18 (p-value = 5.3·10^-3^).

When D = 75 K, we obtained 454 two-gene, 79 three-gene, 28 four-gene, 4 five-gene and 1 six-gene clusters. In total, 35% of the testis-specific genes are in these clusters. By incorporating the variance of gene density in different regions on a chromosome, we conclude that 10 two-gene, 2 three-gene and 3 four-gene clusters are significant with p-value less than 4.1·10^-2^, 3.1·10^-2 ^and 4.9·10^-2 ^respectively. The most significant one is a two-gene cluster on chromosome 16 (p-value = 5.2·10^-3^).

When D = 100 K, we obtained 463 two-gene, 114 three-gene, 46 four-gene, 7 five-gene and 2 six-gene clusters. In total, 41% of the testis-specific genes are in these clusters. By incorporating the variance of gene density in different regions even on the same chromosome, we conclude that 12 two-gene, 4 three-gene and 1 four-gene clusters are significant with p-value less than 4.9·10^-2^, 4.8·10^-2 ^and 3.2·10^-2 ^respectively. The most significant one is a two-gene cluster on chromosome 16 (p-value = 9.2·10^-3^).

We also observed that there are more significant gene clusters on chromosomes 5, 7, 12 X than any other chromosomes.

## Discussion

It seems that in most eukaryotes, the transcription factor machinery is sufficient for ensuring co-regulation so that co-localization of genes in a genome is not necessary. However, there could be some degree of selection for keeping co-regulated genes in clusters on a chromosome, for instance, to make them available to transcription more efficient as a group.

### Gene clusters due to tandem duplication

Clustering of co-expressed genes could be due to tandem duplication. Tandem duplication and subsequent divergence of amplified copies might result in an array of paralogous genes that may retain common regulatory element. Such a type of clustering is exemplified by the Hox gene family [14]. From the testis-specific gene representation profile, we can observe that besides co-expressed adjacent gene pairs, there are large-scale clusters on chromosomes. To determine to what degree tandem duplication contributes to the clustering of testis-specific co-expressed genes, we analyzed cluster distribution after removal of duplicated genes. To identify duplicated testis-specific genes, we performed an all-against-all BLAST homology search using default BLASTN settings. (See the method part for details.) In total, 240 duplicated genes were identified and removed from the list of the testis-specific genes (see Table 3 for details).

**Table 3:** Testis-specific gene distribution before and after removal of tandem duplications.

**Chromosome**	**Number of genes**		**Difference**
		
	**Before removal**	**After removal**	
**1**	249	234	15
**2**	298	280	18
**3**	223	209	14
**4**	230	220	10
**5**	253	232	21
**6**	210	201	9
**7**	229	211	18
**8**	220	205	15
**9**	217	207	10
**10**	223	207	16
**11**	250	239	11
**12**	171	162	9
**13**	179	168	11
**14**	213	199	14
**15**	142	136	6
**16**	128	119	9
**17**	155	145	10
**18**	104	94	10
**19**	109	106	3
**X**	113	105	8
**Y**	12	9	3
**Total**	3928	3688	240

In the most of chromosomes except for chromosomes 2, 5, 7, 10, no more than 15 duplicated genes were identified. On chromosomes 2, 5, 7, 10, we identified 18, 21, 19, 16 duplicated genes respectively. Since, for each of distance threshold value (25 K, 50 K, 75 K and 100 K), we obtained more than 300 gene clusters, we conclude that duplication has no obvious effect on the positional clustering of testis-specific genes in the mouse genome.

### Non-duplicated clusters of genes

Eukaryotic genes have not only promoters, but also one or more enhancers. An enhancer is a DNA sequence that influences transcription of neighboring genes. Enhancers are different from promoters in their ability to act over thousands of base pairs upstream or downstream of a gene. While a promoter is responsible for initiating low levels of transcription and determining the transcription start site, enhancers are responsible for increasing or "enhancing" transcription levels, as well as for regulating cell- or tissue-specific transcription.

There are presently two models for non-duplicated gene clustering. One is the incidental expression mode. In this model, co-regulation within an expression neighborhood is due to incidental interactions between transcriptional enhancers and promoters [16]. When transcription factor binds at the appropriate sites and activates nearby genes together with the target gene, a group of nearby genes tend to express together. In this case sites that bind strong transcriptional activators should create new neighboring co-expressed genes.

An alternative model of clustering is that gene clusters may correspond to regions of active chromatin. This explanation is called the chromatin domain model. In mammalian genomes, gene clusters are on a much larger scale (Mb rather than 5–20 kb) and may involve long-range chromatin interactions [18,19]. The structure of the chromatin domain model assumes that opening of the chromatin of an entire cluster depends on activation of a target gene within the cluster.

Our findings can most be explained as regulation at the level of chromatin structure for the following reasons. First, the regions showing similarities in expression are quite large; each gene presumably has its own core promoter. Second, one or two genes in a group often displays a high level of differential expression. If the chromatin in a region that contains many genes was 'opened' so that a single target gene could be expressed, it might increase the accessibility of the promoters and enhancers of other genes to transcriptional machinery, leading to corresponding increases in their expression. Such an effect could account for the observations we have made. Although the organization of neighborhoods along a chromosome indicates that there must be cis-acting structures, no known structure correlates with the blocks. The structure basis of positional gene clustering is still under investigation.

After removal of tandemly duplicated genes, significant clusters of co-expressed testis-specific genes were still found on all the chromosomes. However, the mechanism responsible for this observation is still unknown.

## Conclusion

We revealed the positional clustering of testis-specific genes in the mouse genome by using EST profiling. Our analysis implies that the mouse genome is enriched with clusters of testis-specific genes. A significant trend for large clusters (at least 3 genes) was discernible. Positional clustering of co-expressed, non-homologous genes has also been reported in nematode [3] and in mammals [6]. This indicates that positional clustering is common in higher eukaryotes. Clustering of tissue-specific genes suggests that there exists some higher order in regulation of gene expression, most probably due to the chromatin domain.

From this fascinating starting point we expect further insight into the significance of gene clustering and the mechanisms that generate them as more genomes are sequenced and more expression patterns are studied in the coming years.

## Methods

### Testis expressed gene models

A total of 60,770 full-length cDNA sequences are available in the Fantom (Funtional Annotation of Mouse) 2.1.1 database. These sequences were aligned back to the mouse genome using BLAT [21] (a program that specializes in the alignment of EST/cDNA sequences to genomic sequences). Overlapping alignments were assembled to produce a complete gene model. This resulted in 34,402 genes. The 3,928 testis-specific genes were identified on the basis of having the keyword "testis" but not the keyword "embryo" in the tissue type information associated with the library.

### Removal of tandem repeats

Clusters of co-expressed genes could be due to tandem duplication. To remove effect of local duplication, we did an all-against-all BLAST search on all 3,928 testis-specific genes. As a result, 240 duplicated genes were identified on the basis of an expectancy value less than 10^-50 ^as proposed by Friedman and Hughes [20]. This results in the final 3,688 testis-specific genes used for our statistical analysis.

### Statistic analysis

#### The neighborhood model

Recall that we define clusters using the distance between two neighboring testis-specific genes along the chromosome. Two testis-specific genes are in a cluster if and only if there is a series of testis-specific genes between them such that the distance between two consecutive genes in the series is less than a specified threshold (*D*).

To incorporate the variance of gene density in different regions on a chromosome, we divide a chromosome into segments of length *L *(2000 Kbp here) so that genes on such a segment are roughly uniformly distributed and analyze the significance of a cluster within the segment according to the number *N *of all the genes (not just testis-specific genes) in the segment and the values of parameters *L *and *D*.

Since we assume the (start) position of a gene is uniformly distributed in a segment of length L, it falls in an interval (*x*, *x *+ *D*) in the segment with probability *D*/*L*. Thus, the number of genes that fall in this interval has a binomial distribution with mean *ND*/*L*. In our case, *D*/*L *is smaller than 0.1. This distribution is approximately Poisson with mean *ND*/*L*. Here we use this approximation in our analysis. The Poisson approximation implies that the number of the genes that falls in a randomly chosen interval of length *D *has a Poisson distribution with mean *m *= *ND*/*L*, and so the probability that at least one gene falls in this interval is 1 - *e*^-*m*^.

Suppose *g*_1_, *g*_2_, …, *g*_*n *_form a cluster in the segment. Then, all the distances of two successive genes are less than *D*. Thus, the number of genes in a cluster minus one has a geometric distribution with p = 1 - *e*^-*m *^(see page 10, [22]). This implies that the probability that a cluster has n genes is (1 - *p*) *p*^*n*-1^, *n *= 0, 1, 2, …. Hence, the p-value of a cluster with *n *genes is the probability that a cluster has more than n genes, which is *p*^*n*^.

In our analysis, *D *= 25 K, 50, 75 K, 100 K. Actually, the distance between two successive testis-specific genes in the most of observed clusters is much smaller than D in each case. Hence, the real p-value for each cluster could be much smaller than one reported in the result section.

#### Adjacent gene clustering

To remove the effect of non-uniform distribution of genes on analysis, we adopt an idea from order statistics. Here, we focus on the position rank of a gene rather than its specific position by ordering all the genes according to their positions on a chromosome. We also treat the set of the testis-specific genes and the set of all the other genes as two types of identical objects. In this way, a specific gene distribution on a chromosome is modeled as a 0–1 string in which 0 represents a test-specific gene and 1 a non-testis-specific gene.

Assume there are *M *genes in a chromosome and *T *of them are testis-specific genes, where *T *≤ *M*. Then, there are  possible gene arrangements on the chromosome and the number of arrangements with *r *adjacent testis-specific gene clusters is . Thus, the probability that a random arrangement has *r *adjacent testis-specific gene clusters is



Using above formula, we can obtain that the mean number of adjacent testis-specific gene clusters in a random arrangement is



and the standard deviation is . Take chromosome 4 for example. We have *M *= 2060, *T *= 220, we obtain that the mean number is 196.6 and the standard deviation is 4.4. However, we only observed 182 adjacent gene clusters (including singletons) on the chromosome.

## Authors' contributions

BL and QL carried out the software development, sequence analysis, and the design of the study. LXZ conceived of the study, performed the statistical analysis, and participated in its coordination. All authors read and approved the final manuscript.
